# Effectiveness of a structured pharmaceutical review of pharmacotherapeutic plans to reduce drug-related problems in nursing homes: a study protocol of a pragmatic quasi-experimental trial

**DOI:** 10.3389/fphar.2025.1522650

**Published:** 2025-07-11

**Authors:** Cecília Campabadal-Prats, Enric Aragonès, Marta Romeu, Clara Salom-Garrigues, Ferran Bejarano, Francisco Martín Luján, Laura Canadell

**Affiliations:** ^1^ Atenció Primària Camp de Tarragona, Institut Català de la Salut, Tarragona, Spain; ^2^ Institut Universitari d’Investigació en Atenció Primària Jordi Gol (IDIAPJGol), Barcelona, Spain; ^3^ Unit of Research, Atenció Primària Camp de Tarragona, Institut Català de la Salut, Tarragona, Spain; ^4^ Unit of Pharmacology, Department of Basic Medical Sciences, Faculty of Medicine and Health Sciences, Universitat Rovira i Virgili, Reus, Spain; ^5^ Hospital Joan XXIII, Institut Català de la Salut, Tarragona, Spain

**Keywords:** nursing homes, polypharmacy, adverse drug event, pharmaceutical care, drug related problems (DRP)

## Abstract

**Background:**

Patients living in nursing homes have increased aging, comorbidity, spending on health resources and mortality. High drug use is associated with an increased risk of falls, disability, and death. It is estimated that 20%–50% of inappropriate medications are consumed in the elderly.

**Objective:**

This abstract outline the study protocol for evaluating the effectiveness of a pharmaceutical intervention designed to optimize medication use among nursing home residents, with a particular focus on reducing drug-related problems (DRPs) and minimizing polypharmacy.

**Hypothesis:**

The pharmaceutical intervention led by a primary care pharmacist and based on systematically reviewing the pharmacotherapeutic plans of patients admitted to nursing homes will effectively improve the quality and safety of treatment plans.

**Methodology:**

Pre-post, quasi-experimental intervention study with a control group and prospective 3-month follow-up of a cohort of patients in nursing homes. The intervention consists of a clinical review of the pharmacotherapeutic plan carried out by the pharmacist and subsequently agreed upon in the pharmacotherapeutic advisory committee.

**Determinations:**

The study variables will be evaluated at baseline and 3 months post-intervention. The outcome variables are drug-related problems and polymedication.

**Statistical analysis:**

Percentage change will be measured before and after the intervention. Descriptive statistics will be performed for quantitative variables such as qualitative and comparison of means and proportions.

**Expected results:**

Decreasing 10% DRPs in terms of number of DRPs per patient as well as the proportion of patients experiencing DRPs. Reducing 10% polymedication in terms of the number of drugs per patient as well as the number of polymedicated patients.

**Applicability and relevance:**

This study will expand the collaboration between the pharmacy and primary care physicians, promoting the continuum of care, strengthening the safety culture, and improving prescribing habits.

**Ethics:**

The protocol was approved by the Research Ethics Committee of the Jordi Gol Primary Care Research Institute (IDIAP), Barcelona, 22/191-EOm.

**Trial registration:**

clinicaltrials.gov, NCT05944328 Registered.

## Introduction

Drug-related problems (DRPs)—including potentially inappropriate medications (PIMs), medication errors (MEs), and adverse drug events (ADEs)—represent a major and avoidable public health issue (Meyer-Massetti et al., 2018). DRPs are considered the most prevalent cause of iatrogenesis worldwide, particularly among frail patients. There is robust evidence indicating that DRP-related morbidity is a largely preventable health burden (Ruiz-Ramos et al., 2021).

Approximately 38% of emergency department visits are associated with an DRP, of which more than 70% are considered preventable (Baena et al., 2014; Patel and Zed, 2002). Additionally, DRPs account for 5%–10% of hospital admissions and 21% of readmissions (Nivya et al., 2015). Risk factors for DRPs include advanced age, polypharmacy, comorbidities, anticoagulant use, and cognitive impairment, placing individuals over 65 years at particularly high risk (Zhou et al., 2018).

Polypharmacy may be defined qualitatively as the use of more medications than are clinically appropriate, and quantitatively by a numerical threshold. It is commonly defined as the chronic use of five or more medications, while the risk increases significantly with ten or more prescribed drugs, warranting closer clinical attention (Duerden et al., 2013; Gnjidic et al., 2012). Several studies have demonstrated a strong association between the number of medications and the likelihood of ADEs, with the risk rising from 6% with two drugs, to 50% with five, and nearly 100% with eight or more medications (Chumney and Robinson, 2006).

A recent study has linked polypharmacy with an increased risk of falls, with the prevalence of fall risk being twice as high in patients taking 10 or more medications, particularly with antipsychotics and sedative-hypnotics (Turegano et al., 2019). Residents in nursing homes consume approximately four times more medications than community-dwelling individuals with similar clinical profiles (Walley and Scott, 1995). This high level of prescribing often results from the uncritical application of clinical practice guidelines without individualizing therapeutic decisions based on the patient’s benefit–risk profile (Holmes et al., 2006).

Consequently, DRPs—particularly PIMs—are highly prevalent in nursing home residents (Leguelinel-Blache et al., 2020). A PIM is defined as a prescription in which the potential risks outweigh the expected benefits, especially when safer alternatives exist (Delgado-Silveira et al., 2015). It is estimated that approximately 40% of prescriptions in nursing home residents may be inappropriate (Leguelinel-Blache et al., 2020). Various studies have found that 15%–50% of residents are exposed to at least one PIM, with an average of 2.2–4 DRPs per patient (Forsetlund et al., 2011).

Vink et al. (2011) found that pharmacists were able to identify DRPs that other healthcare professionals did not detect. Most DRPs were related to suboptimal therapies or unnecessary medications (Monzón-Kenneke et al., 2021). Comprehensive medication review is a patient-centered approach aimed at optimizing drug therapy and improving clinical outcomes by ensuring that each medication is indicated, effective, and safe for the patient’s current condition (Roth McClurg et al., 2024).

In recent years, pharmacist-led medication review programs have been implemented in nursing homes. Interventions conducted by multidisciplinary teams (physicians, nurses, and pharmacists) have shown reductions in healthcare costs and iatrogenic risks, although results have been heterogeneous and sometimes difficult to interpret. A recent meta-analysis suggests that pharmacist-led deprescribing programs in nursing homes can reduce the prevalence of PIMs by 59% (Kua et al., 2019). Another meta-analysis found that pharmaceutical interventions in nursing homes resulted in a 43.8% reduction in the incidence of falls (Lee et al., 2019).

Aligned with the principles of the Catalan national strategy for primary and community care 2016–2020 (Generalitat de Catalunya, 2015), the Interdepartmental plan for health and social care (Departament de Salut, 2019) was developed to provide integrated, person-centred care to nursing home residents. This plan proposes an efficient pharmaceutical care model that enhances medication safety, quality of life, and health outcomes, with the explicit goal of integrating the pharmacist into the multidisciplinary care team.

As part of the Program for chronic care management developed by the Catalan department of health (Departament de Salut, 2015), the Basic guide for medication management in chronic patients was published (Departament de Salut, 2013). This guide outlines a methodology for treatment optimization through patient-centred, multidisciplinary medication review procedures involving reconciliation, review, deprescribing, and adherence strategies.

In this context, the Primary care directorate of the Camp de Tarragona region (Catalan health institute) oversees 24 nursing homes linked to 20 primary care teams, serving a total of 1,928 residents in 2022. Within this policy framework, a pharmaceutical care program was launched in nursing homes with the aim of reducing DRPs and improving medication safety.

This abstract presents the study protocol, which aims to evaluate the effectiveness of a pharmacist-led intervention to optimize medication use in nursing home residents.

### Hypothesis

This study hypothesizes that a pharmacist-led intervention involving the systematic review of pharmacotherapeutic plans in nursing home residents will improve the quality and safety of their medication regimens.

## Methods

### Objective

The primary objective of this study is to evaluate the effectiveness of a structured pharmaceutical intervention based on the review and optimization of pharmacotherapeutic plans to improve their appropriateness for individuals residing in nursing homes.

Specifically, the study aims to determine, over a 3-month period, the effectiveness of the program in (Meyer-Massetti et al., 2018): reducing drug-related problems (DRPs), as measured by the number of DRPs per patient and the proportion of patients with DRPs, and (Ruiz-Ramos et al., 2021) reducing polypharmacy, in terms of the average number of medications per patient and the number of patients exposed to polypharmacy.

### Design

**FIGURE 1:**
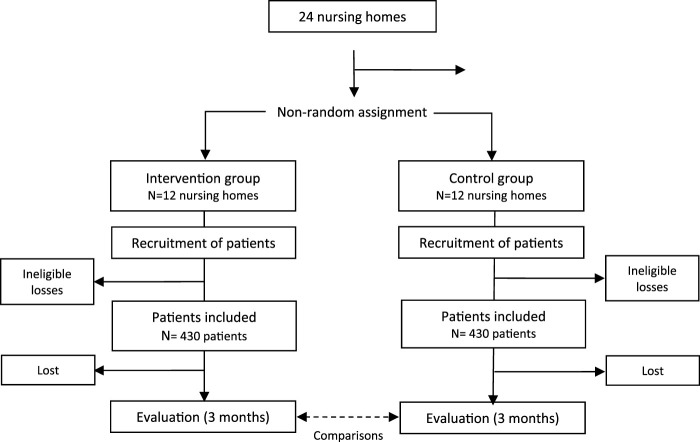
Study development algorithm: centre allocation, sampling and monitoring.

This is a quasi-experimental study using a non-randomized cluster assignment of patients (nursing homes) into two groups: (a) an intervention group receiving a structured review and optimization of pharmacotherapeutic plans, and (b) a control group receiving usual care based on standard clinical practice (Figure 1).

### Study location and sample

The study will be conducted in the 24 nursing homes served by primary care teams from the Primary care directorate of Camp de Tarragona (Catalan health institute).

Inclusion criteria: individuals aged 65 years or older, on pharmacological treatment with at least one medication, for a period longer than 3 months. Exclusion criteria include: patients hospitalized at the time of the review, those in end-of-life care, those for whom participation may be deemed potentially harmful by the responsible physician, and patients not covered by the public healthcare or pharmacy system.

### Assignment to study groups

Clustering was defined at the level of the nursing home to minimize the risk of contamination, as residents within the same facility are typically treated by the same medical and care teams. Assigning entire centers to either the intervention or control group also facilitated the coordinated implementation of the structured medication review process and aligned with the organizational routines of the participating facilities.

Assignment to either the intervention or control group will be based on convenience within the context of implementing the pharmaceutical review program in the study area. The first 12 nursing homes in which the program is implemented will be allocated to the intervention group, with the remaining nursing homes forming the control group. The comparability of the groups will be ensured in terms of nursing home characteristics (geographical location, size, organizational and healthcare features) and patient profiles (sociodemographic and clinical variables.

### Intervention

The intervention involves a systematic and structured review of patients’ pharmacotherapeutic treatment plans to identify problems or risks associated with their medication regimens and propose changes or improvements. This procedure is part of the standard service portfolio of the regional healthcare authority and is conducted by pharmacists from the territorial Primary Care Pharmacy Unit. The intervention is based on the protocol outlined in the document “Rational use of medicines. Basic medication management in chronic patients: Reconciliation, review, desprescription, and adherence (Departament de Salut, 2013).

The review process includes two phases. In the first phase, individual pharmacological treatment plans are reviewed to identify DRPs, which are classified into four types: indication-related, appropriateness, effectiveness, and safety issues. Indication-related DRPs include medications. Without a current indication or missing necessary medications.

Appropriateness-related DRPs assess dosage, frequency, regimen, and duration, as well as the suitability of medications based on age, renal and hepatic function, and other clinical parameters. To identify potentially inappropriate medications by age group, we used the reference “Potentially inappropriate medications for the elderly” (Servei Català de la Salut, 2020), which integrates the Beers Criteria (American Geriatrics Society Beers Criteria Update Expert Panel, 2019), STOPP-START criteria (O'Mahony et al., 2015), Priscus List (Holt et al., 2010), and EU-PIM List (Fick et al., 2015), providing justification, recommendations for change and the criteria on which these recommendations are based. Effectiveness-related DRPs assess therapeutic outcomes in relation to clinical goals and identify instances of overtreatment or the need for de-intensification based on the patient’s health status.

Safety-related DRPs include therapeutic duplications, contraindications, adverse effects, allergies, drug interactions, or insufficient laboratory monitoring.

In the second phase, pharmacists issue proposals to optimize pharmacotherapy in response to identified DRPs. Recommendations may include discontinuation, substitution, therapeutic equivalents, initiation of new treatments, dosage or frequency adjustments, or enhanced monitoring.

These recommendations are then evaluated and agreed upon by a committee composed of the responsible physicians and the pharmacist. Consensus-based proposals are subsequently discussed with the patient or their legal representative, leading to a shared decision. Recommendations may be accepted or rejected.

### Usual care (control group)

In the control nursing homes, pharmacological treatments will be managed following standard procedures. Typically, primary care physicians only authorize and prescribe treatments proposed by nursing home physicians, with limited involvement in ongoing medication management. However, they have access to electronic clinical decision support tools aimed at improving prescribing quality. These include the self-audit tool which detects therapeutic duplications, polypharmacy, contraindications, and inappropriate treatment durations- and the PREFASEG (Safe pharmacological prescription) module, which alerts prescribers to potential interactions, contraindications, and redundancies when initiating new therapies.

### Measurement

Data were collected at baseline and at 3 months using the electronic prescription module of each patient’s electronic health record. The evaluation was conducted by the pharmacist implementing the intervention, who was not blinded to the study group allocation of nursing homes and patients (Table 1).

➢ Outcome variables

**TABLE 1:** Variables and measuring instruments.

Variable	Instrument	Application time	
		Basal	3 months
Sociodemographic variables	Questionnaire *ad hoc*	X	
Age			
Sex			
Length of time living in the nursing home			
Pharmacotherapeutic plan			
Description of the pharmacotherapeutic plan	ATC classification^a^	X	
Description and classification of the DPRs^b^ detected	Classification	X	
Clinical			
Comorbidity			
*Physical comorbidity*	Checklist	X	
*Psychiatric comorbidity*	Checklist		
*Global indicator*	*Charlson index*	X	
Complex Chronic Patient^c^	Checklist	X	
Advanced Chronic Care Model^c^	Checklist	X	
Functional capacity	Barthel index	X	
Cognitive status	SPMSQ^d^ Pfeiffer		
Clinical Results			
Drug related problems (DRP)^b^	Questionnaire *ad hoc*	X	X
*Number of DR P* ^b^ *per patient*			
*Proportion of patients with DRP*			
Polymedication	Questionnaire *ad hoc*	X	X
*Number of drugs a patient receives simultaneously*			
*Number of polymedicated patients*			
Results of the pharmaceutical review (only in the intervention group)			
Proposals for change	Questionnaire *ad hoc*	X	
Proposals implemented	Questionnaire *ad hoc*		X

^a^ ATC: anatomical therapeutic chemical classification system.

^b^ DRP: drug related problems.

^c^ According to the criteria of *Bases per a un model català d’atenció a les persones amb necessitats complexes. Conceptualització i introducció als elements operatius. Generalitat de Catalunya, 2017.*

^d^ SPMSQ: short portable mental status questionnaire.

Drug-Related Problems (DRPs) were quantified as total number, DRPs per patient, and the proportion of patients with at least one DRP. DRPs were classified into four categories: indication, appropriateness, effectiveness, and safety (Table 2).

**TABLE 2:** List and classification of drug-related problems (DRP) considered in this study.

Category	DRP
Indication	- Medically unnecessary/not indicated/not appropriate for the health problem - Failure to prescribe a necessary medicine
Adequacy	- Medically inappropriate - Dose higher or lower than correct dosage - Incorrect administration frequency - Wrong drug form - Duration too long or too short
Effectiveness	- Failure to achieve the therapeutic goal - The goal is achieved but the intensity of the pharmacological treatment needs to be reduced - Not the most effective alternative based on evidence and/or clinical practice guidelines
Safety	- Allergy or adverse effect - Contraindication - Therapeutic duplication - Drug- drug interaction - Lack of analytical controls

This classification is based on the position statement of the Spanish Society of Primary Care Pharmacists (*Sociedad Española de Farmacéuticos de Atención Primaria*) (Socied ad Española de Farmacéuticos de Atención Primaria SEFAP, 2022).

Polymedication was measured as the number of medications taken concurrently by each patient and the number of polymedicated patients. Polypharmacy was defined as the chronic use of ≥10 medications (Duerden et al., 2013; Gnjidic et al., 2012). Topical treatments, eye drops, occasional treatments, and those used for acute conditions were excluded from this count. Fixed-dose combinations were counted according to the number of active ingredients. Multiple dosages of the same active ingredient were counted as one drug.

➢ Secondary variables

Sociodemographic variables such as age and sex were collected. Regarding the pharmacotherapeutic plan, the number and classification of DRPs identified (by indication, appropriateness, effectiveness, or safety) were recorded.

Clinical variables included physical comorbidity, such as the presence of chronic conditions like diabetes, cardiovascular disease, respiratory disease, dementia, and cancer. Psychiatric comorbidity included diagnoses of depression, anxiety, psychotic disorders, and substance use disorders.

Comorbidity was also quantified using the Charlson Comorbidity Index (Charlson et al., 1987), which integrates age and 19 clinical conditions to provide a summary score correlated with 1-year and 10-year survival.

To assess chronic care complexity, we recorded the number of patients identified as Complex chronic patients (PCC) or as part of the Advanced chronic care model (MACA) (Departament de Salut, 2017). PCCs are defined by clinicians as having complex clinical management needs due to multimorbidity, a polypharmacy, clinical instability, resource use, and age extremes. MACA patients are those with a limited life expectancy and palliative care needs.

Functional status was assessed using the Barthel Index (Mahoney and Barthel, 1965), which evaluates independence in activities of daily living on a scale from 0 (total dependence) to 100 (total independence), categorized as severe (0–50), moderate (51–75), or mild/no dependency (76–100).

Cognitive status was assessed using the validated Short portable mental status questionnaire (SPMSQ) by Pfeiffer (Martínez de la Iglesia et al., 2001), composed of 10 items scored from 0 to 10. Cognitive impairment was classified as: none (0–2), mild (Baena et al., 2014; Patel and Zed, 2002), moderate (Nivya et al., 2015; Zhou et al., 2018; Duerden et al., 2013), or severe (≥8).

In the intervention group, pharmacist recommendations were categorized as (Meyer-Massetti et al., 2018): drug discontinuation (Ruiz-Ramos et al., 2021), drug substitution (Baena et al., 2014), therapeutic equivalent change (Patel and Zed, 2002), treatment initiation (Nivya et al., 2015), close monitoring (Zhou et al., 2018), dose adjustment, or (Duerden et al., 2013) frequency adjustment. The number of recommendations implemented within 3 months was also recorded.

## Statistical methods

### Sample size

Based on prior observations and published studies, we assume that the proportion of patients with drug-related problems (DRPs) will be 50% in the control group (Vink et al., 2011; Costa et al., 2016; Garin et al., 2021). We expect to detect an absolute reduction of 10 percentage points in the prevalence of medication-related problems, from 50% in the control group to 40% in the intervention group. This difference is considered clinically meaningful, as even a modest reduction in such problems may lead to significant improvements in patient safety and care quality in the nursing home setting.

The sample size is calculated to account for the cluster design of the study, using the GRANMO calculator (https://www.datarus.eu/en/applications/granmo/). Assuming an alpha risk of 0.05 and a power of 0.80 in a two-sided test to detect a difference between proportions of 0.50 in the control group and 0.40 in the intervention group, a total of 24 clusters (12 per group) with 37 participants per cluster is required, including an estimated 10% loss to follow-up. A low intraclass correlation coefficient (ICC) of 0.001 is assumed. In the absence of published ICC estimates for this setting, we adopt a conservative value consistent with other studies involving individual-level binary outcomes with limited clustering. If the actual ICC or the number of participants per cluster differs substantially from expectations, we will conduct a sensitivity analysis to assess the potential impact on statistical power and precision.

Participants who die during the follow-up period will be considered lost to follow-up and excluded from outcome analyses. This expected attrition has been accounted for in the sample size calculation, which includes an estimated 10% loss due to mortality and other causes of dropout.

### Statistical method

The main analyses will be by intention to treat, taking into account the patients in each group according to the initial assignment to the study groups, regardless of the compliance with the program by the patient or the responsible physicians, or any other pharmaceutical adequacy intervention that the physicians or patients in the control group may receive.

We will first describe the baseline characteristics of the sample and compare the intervention and control groups to assess baseline comparability. Although the intervention is mainly directed at the prescribing physician through recommendations or suggested changes, outcomes will be assessed at the individual patient level. We will account for potential non-independence of observations arising from patients being managed by the same physician (cluster effect).

Primary outcome variables include: number of DRPs per patient, proportion of patients with DRPs, number of medications per patient, and proportion of polymedicated patients. The effects of the intervention on dichotomous outcomes will be assessed using multilevel mixed-effects logistic regression models (cluster-adjusted), with the odds ratio (95% CI) of the intervention group *versus* control serving as the measure of effect. For continuous variables, we will use linear mixed-effects regression models (cluster), calculating differences between adjusted means (95% CI) between the intervention and control groups. Statistical significance will be set at p < 0.05.

### Ethical aspects

This is a pragmatic clinical trial conducted under real-world clinical practice conditions. The pharmaceutical intervention under study is part of the primary care pharmaceutical services.

Patient data will be extracted from the Electronic health record by the Department of information systems. The principal investigator (PI) will work with a dataset derived from this extraction, in which patient information will be pseudonymized. Pseudonymization process will be carried out by the Department of information systems. This department will generate and send a code–patient identity correspondence document to the responsible clinician, exclusively for the purpose of facilitating the shared decision-making process. PI will not have access to the document linking patient identity to the pseudonymized codes, ensuring compliance with data protection and confidentiality standards. Portfolio and is being evaluated during its implementation process in routine clinical practice.

In all cases, it is the responsibility of the treating physician to evaluate the applicability of the recommendations generated by the medication review, based on clinical judgment and in consultation with the patient. As a safeguard against potential harm, a criterion for exclusion was that the physician considered participation potentially detrimental for the patient. Patients in the control group facilities received the pharmaceutical intervention after the study follow-up period, according to the planned implementation process.

The study was designed in accordance with the Good research practice guidelines in health sciences of the Catalan health institute (Institut Català de la Salut, 2010), the principles of the Declaration of Helsinki (2013 revision), and applicable regulations.

The protocol was reviewed and formally approved by the Clinical research ethics committee (CEIC) of IDIAP Jordi Gol (Barcelona), including the formal exemption from informed consent for patients.

## Discussion

This is a pragmatic study that integrates an evaluation framework into the implementation process of a new medication review and optimization program in nursing homes. Although the intervention is well-established and supported by scientific evidence, the main goal of this study is to assess its utility and effectiveness in routine clinical practice.

A novel aspect of the program is the expanded role of the clinical pharmacist as a support and advisor to general practitioners in identifying drug-related problems and proposing solutions. This approach aligns with healthcare improvement initiatives in nursing homes, involving a redefinition of professional roles and the promotion of multidisciplinary teamwork (Departament de Salut, 2019).

This study will have certain limitations that should be considered when interpreting the results. One of the main concerns will be the non-random allocation of care homes to the intervention and control groups, which was based on convenience and collaboration feasibility within the framework of the local pharmaceutical review program. Although this approach is justified for practical reasons, it may introduce a potential risk of selection bias. To minimize this, we will ensure that the two groups are comparable at baseline in terms of care home characteristics (e.g., geographical location, size, and organizational structure) and patient-level variables (e.g., sociodemographic and clinical profiles, as well as the quality of pharmacotherapeutic plans). Any residual imbalances will be addressed through appropriate statistical adjustments during the analysis.

The fact that the assessment is neither blind nor independent introduces a potential risk of information bias, as the pharmacists responsible for implementing the intervention also carry out the outcome assessments in the intervention group. However, this risk is mitigated by the fact that outcome data will be obtained directly and systematically from the electronic health records, based on structured variables that are not modifiable by the assessors. Due to resource limitations, it is not feasible to separate intervention and assessment roles, and this potential bias should be considered when interpreting the study results.

According to a meta-analysis evaluating pharmacist-led interventions within multidisciplinary teams (physician, nurse, pharmacist) in nursing homes, such interventions led to a reduction of 2.2 DRPs (0.28–4.12; I^2^ = 44%, p = 0.02) based on the MAI criteria, a mean reduction of 1.9 medications, and an intervention acceptance rate of 69.8% (Lee et al., 2019).

The results of this study will inform the real-world utility and feasibility of the evaluated intervention and its implementation process, including the evolving role of the primary care pharmacist within a multidisciplinary team. This information will be valuable in refining the medication review program and optimizing its practical implementation.

## Data Availability

The original contributions presented in the study are included in the article/supplementary material, further inquiries can be directed to the corresponding author.
